# Discovery of a novel target for renal cell carcinoma: transglutaminase 2

**DOI:** 10.1038/cddis.2016.99

**Published:** 2016-04-21

**Authors:** J H Kang, S-H Lee, S-Y Kim

**Affiliations:** 1 Cancer Cell and Molecular Biology Branch, Division of Cancer Biology, Research Institute, National Cancer Center, Goyang, Gyeonggi-do, Korea

Instability of p53 in renal cell carcinoma (RCC) has not been previously associated with mutations, because 96% of clear-cell RCC samples in the database were present with the p53 wild type.^1^ However, inhibition of a major p53 regulator in humans doubles minute 2 homolog (HDM2) induced cell cycle arrest but not cell death in RCC.^2^ Therefore, overcoming p53 instability is a major issue in RCC treatment.

Recently, our group^3^ and others^4^ have found that the expression level of transglutaminase 2 (TGase 2, E.C. 2.1.2.13) is generally elevated in most RCC cell lines, which deplete p53 into aggregates in the autophagosome, resulting in p53 depletion through autophagy.^3^ This p53 instability by TGase 2 regulation allows tumor cells to evade apoptosis and grow remarkably.

TGase 2 is an enzyme that catalyzes an isopeptide bond between protein glutamine and lysine residues, resulting in a covalent cross-link.^5^ In normal physiological conditions, TGase 2 contributes toward regulating apoptosis from intruders or damages, including biological, chemical and physical challenges.^6^ However, TGase 2 knockout mice display impaired autophagy under starvation,^7^ although TGase 2 knockout mice present with an otherwise normal life.^8, 9^ Cancer cells adopt TGase 2 mediated autophagy for survival.

TGase 2 competes with HDM2 for binding to p53; promotes autophagy-dependent p53 degradation in RCC cell lines under starvation; and binds to p53 and p62 simultaneously without ubiquitin-dependent recognition of p62. The bound complex does not have cross-linking activity. A binding assay using a series of deletion mutants of p62, p53 and TGase 2 revealed that the PB1 domain of p62 (residues 85–110) directly interacts with the β-barrel domains of TGase 2 (residues 592–687), whereas the HDM2-binding domain (transactivation domain, residues 15–26) of p53 interacts with the N-terminus of TGase 2 (residues 1–139).^1^ During translocation of p53 to the autophagosome through TGase 2 binding, cross-linking activity is not needed. This finding is in agreement with the observation that TGase 2 cross-linking activity occurs only in the autophagosome during autophagy.^1, 7^ This suggests that TGase 2 acts as a chaperone of p53 with a cross-linking catalytic activity. This interaction may result in rapid autophagy without consuming energy to tag ubiquitin on p53, as p62 is known to interact with ubiquitinated proteins (see Figure 1). This autophagy process is beneficial in which it supplies building blocks, including degraded p53, for cancer cells.

We recently reported that monotherapy using the TGase 2 inhibitor GK921 in a xenograft tumor model abrogated RCC growth through p53 stabilization.^10^ In addition to the increase in p53 stability due to TGase 2 inhibition, the administration of a DNA-damaging anti-cancer drug such as doxorubicin, remarkably induced apoptosis in RCC cell lines and sensitively reduced tumor volume in a xenograft model. Combination therapy with a TGase 2 inhibitor and a DNA-damaging agent may represent an effective therapeutic approach for treating RCC.

## Figures and Tables

**Figure 1:**
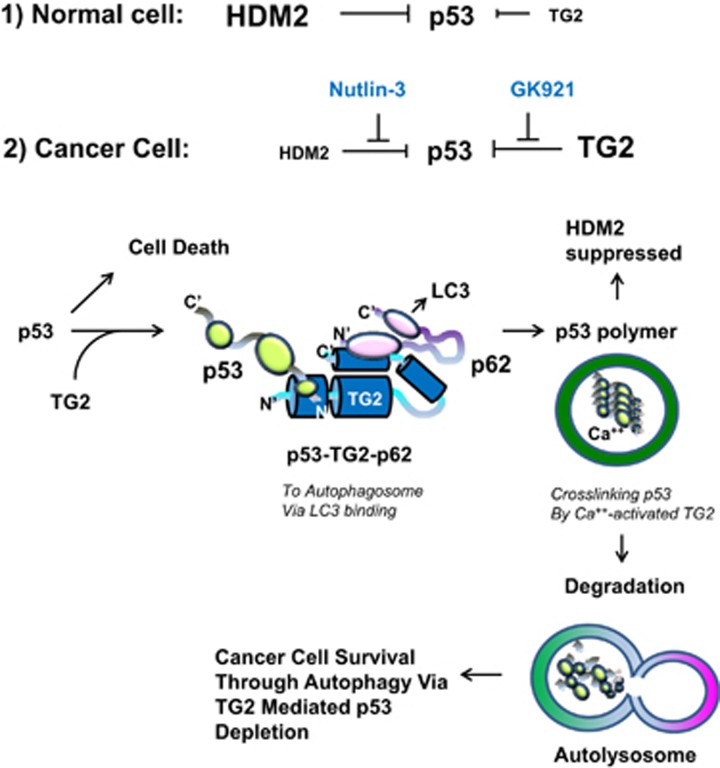
(1) In normal cell, p53 stability depends more on HDM2 regulation due to no induction of TGase 2. (2) In cancer cell, TGase 2 is induced by stress such as hypoxia or starvation. The N-terminus of TGase 2 interacts with the N-terminus of p53 and, simultaneously, the C-terminus of TGase 2 interacts with the N-terminus of p62; as a result, a heterotrimeric complex (p53–TGase 2–p62) is formed. The C-terminus of p62, in the p53–TGase 2–p62 complex, is free and moves the complex to LC3 in the phagophore. When the autophagosome is completed with components of the phagophore, p53 is polymerized by TGase 2 with calcium-dependent activation in the autophagosome. Later, the autophagosome and lysosome are fused into an autolysosome, which degrades all cross-linked materials
